# Highly extensile approach for comminuted ulna coronoid process fractures with mini-plate fixation: a case series of 31 patients

**DOI:** 10.1186/s12891-024-07637-1

**Published:** 2024-07-05

**Authors:** Shi-Cheng Zhou, Sheng-Yu Jin, Qing-Yu Wang, Guang-Kai Ren, Chuan-Gang Peng, Yan-Bing Wang, Dan-Kai Wu

**Affiliations:** Orthopaedic Medical Center, The Second Hospital of Jilin University, No. 218, Ziqiang Street, Nanguan District, Jilin Changchun, People’s Republic of China

**Keywords:** Coronoid process fractures, Highly extensile approach, Mini-plate fixation, Anterior minimally invasive approach, Mayo elbow performance index score

## Abstract

**Background:**

For the treatment of coronoid process fractures, medial, lateral, anterior, anteromedial, and posterior approaches have been increasingly reported; however, there is no general consensus on the method of fixation of coronal fractures. Here, we present a highly-extensile minimally invasive approach to treat coronoid process fractures using a mini-plate that can achieve anatomic reduction, stable fixation, and anterior capsular repair. Further, the study aimed to determine the complication rate of the anterior minimally invasive approach and to evaluate functional and clinical patient-reported outcomes during follow-up.

**Methods:**

Thirty-one patients diagnosed with coronoid fractures accompanied with a “terrible triad” or posteromedial rotational instability between April 2012 and October 2018 were included in the analysis. Anatomical reduction and mini-plate fixation of coronoid fractures were performed using an anterior minimally invasive approach. Patient-reported outcomes were evaluated using the Mayo Elbow Performance Index (MEPI) score, range of motion (ROM), and the visual analog score (VAS). The time of fracture healing and complications were recorded.

**Results:**

The mean follow-up time was 26.7 months (range, 14–60 months). The average time to radiological union was 3.6 ± 1.3 months. During the follow-up period, the average elbow extension was 6.8 ± 2.9° while the average flexion was 129.6 ± 4.6°. According to Morrey’s criteria, 26 (81%) elbows achieved a normal desired ROM. At the last follow-up, the mean MEPI score was 98 ± 3.3 points. There were no instances of elbow instability, elbow joint stiffness, subluxation or dislocation, infection, blood vessel complications, or nerve palsy. Overall, 10 elbows (31%) experienced heterotopic ossification.

**Conclusion:**

An anterior minimally invasive approach allows satisfactory fixation of coronoid fractures while reducing incision complications due to over-dissection of soft tissue injuries. In addition, this incision does not compromise the soft tissue stability of the elbow joint and allows the patient a more rapid return to rehabilitation exercises.

## Background

Fractures of the coronoid process are difficult conditions that are often associated with elbow instability and, without proper management, can result in severe limitation of elbow function [1]. The coronoid process of the ulna is crucial for anterior elbow joint stability [2] and can be injured by axial loading, accompanied by elbow dislocation or subluxation. The incidence rate of coronoid process fractures among patients with dislocation of elbows is between 2% and 15% [3]. A cadaveric series study performed by Closkey et al. [4] showed a significant difference in the posterior instability of elbows with a 50% loss of the coronoid (Regan and Morrey type III) at all flexion angles relative to those with type I or II simulated fractures [5]. More recently, elbow instability with smaller fracture fragments (Regan and Morrey Types I and II) was reported by O’Driscoll et al. [6] and Michael et al. [7], especially when accompanied by a “terrible triad” or posteromedial rotation [8]. Therefore, it is necessary to restore the coronoid process height to ensure postoperative elbow stability.

For the treatment of coronoid process fractures, medial, lateral, anterior, anteromedial, and posterior approaches have been increasingly reported; however, there is no general consensus on the method of fixation of coronal fractures. Moreover, these incisions are associated with postoperative complications, such as dislocation of the elbow, joint stiffness, heterotopic ossification (HO), nerve palsy, and limited range of motion (ROM) of the elbow [9–17]. Furthermore, these approaches provide only a limited visualization for precise fixation of the coronoid process. To effectively overcome these disadvantages, achieve ample exposure, and precisely fix the coronoid process, it is important to explore novel operative approaches to reconstruct the coronoid process.

The present study aimed to investigate the clinical results of treatment of a coronoid fracture with a mini-plate using a minimally invasive anterior highly-extensile approach by assessing clinical, radiological, and cosmetic outcomes.

## Methods

### Study design and participants

**Fig. 1:**
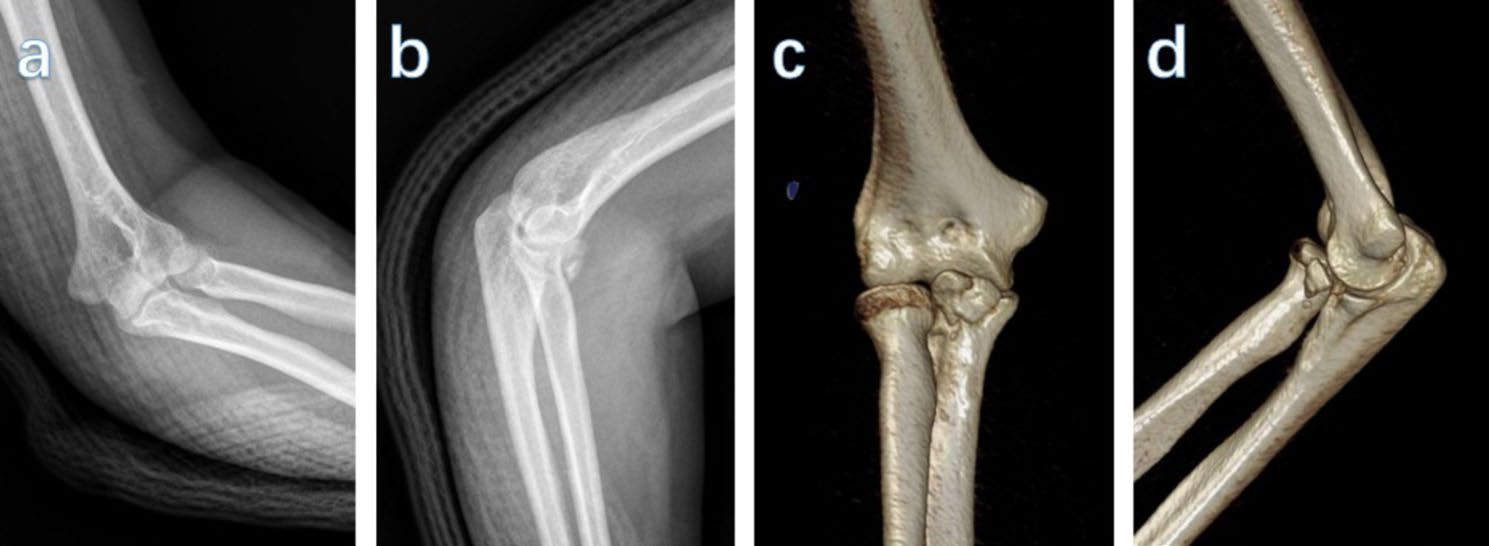
Representative Regan-Morrey type III coronoid fracture. (**a**, **b**) Preoperative radiographs of the coronoid process fracture. (**c**, **d**) Reconstructed three-dimensional computed tomography images of the coronoid process fracture

The ethics committee of our hospital approved this study. Between April 2012 and October 2018, a consecutive series of 32 elbows of 31 patients with a coronoid fracture that underwent mini-plate fixation using a minimally invasive anterior approach was studied (Fig. 1). The following inclusion criteria were applied: adults aged over 18 years and patients with Type III (Regan and Morrey classification [18], OTA/AO type 2U1–B1) coronoid fractures, Type I and II (Regan and Morrey classification [18] OTA/AO type 2U1–B1), coronoid fractures accompanied by a “terrible triad,” or posteromedial rotation instability. The following exclusion criteria were applied: severe injury with an open elbow joint fracture, preexisting brachial artery or median nerve lesion, and patients who could not complete follow-up for more than 12 months. The average age was 44.0 years (range, 23–63), and included 17 women and 14 men. The dominant side was injured in 24 cases (Table 1).

**Table 1:** Review of preoperative statuses of the coronoid process fractures patients

Category	
Number of patients (male/female )	31 patients (14/17)
Number of elbows	32
Age (years)	44.0 (range, 23–63)
Average follow-up period (months)	26.7 (range, 14–60)
Body mass index (kg/m²)	24.25 (range, 21.30–28.50)
ASA^†^	1.7 (range, 1–3)
Regan and Morrey’s classification	
Type I/Type II/Type III	4/23/5
Injured side of the elbow (right/left)	24/8
Associated ligament injury	
MCL	6
LCL	8
Terrible triad	4

American Society of Anesthesiologists (ASA) physical status classification system. MCL, Medial Collateral Ligament. LCL, Lateral Collateral Ligament

### Surgery technique

**Fig. 2:**
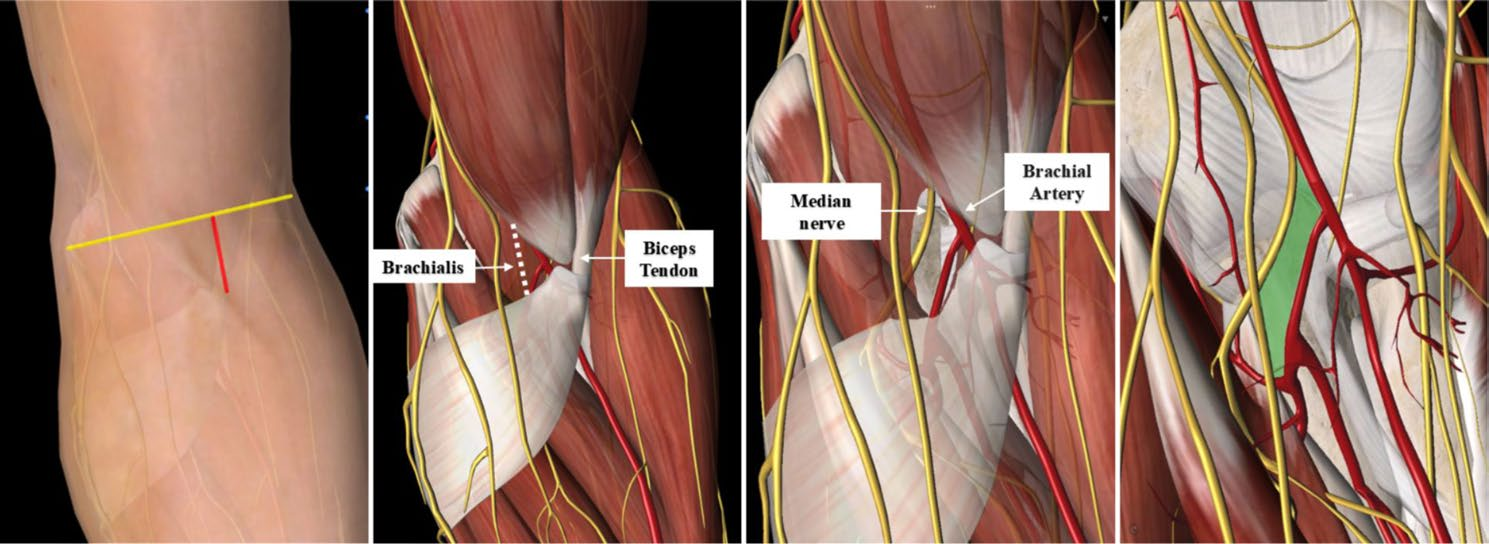
Images showing an anterior minimally invasive approach (the straight longitudinal incision over the coronoid process was made proximally from the main elbow flexion crease and extended 2 cm distally to the elbow flexion crease), and the safe space for insertion was available between the brachial artery and the median nerve

All procedures were performed by the same senior surgeon using a straight anterior, minimally invasive approach. All fractures were fixed with microplates (AZX-LL locking plate fixation system; Besta, Beijing, China). The patient was placed in a supine position, with the affected upper extremity placed on a radiolucent hand table. After anesthesia, the sterile forequarter was prepared and draped, and a tourniquet was tied to the proximal arm. The coronoid process was located approximately 1 cm below the main elbow flexion crease. Landmarks for the skin incision, such as the main elbow flexion crease, were identified. A straight longitudinal incision over the coronoid process was made proximally from the main elbow flexion crease and extended 2 cm distally to the elbow flexion crease. A blunt subcutaneous dissection was performed to cut the subcutaneous tissues layer by layer. After the subcutaneous tissue dissections, the bicipital aponeurosis was exposed. Exposure of the brachioradialis, brachial artery, vein, and median nerve was best achieved by splitting the biceps aponeurosis in line with underlying nerve and artery (Fig. 2).

**Fig. 3:**
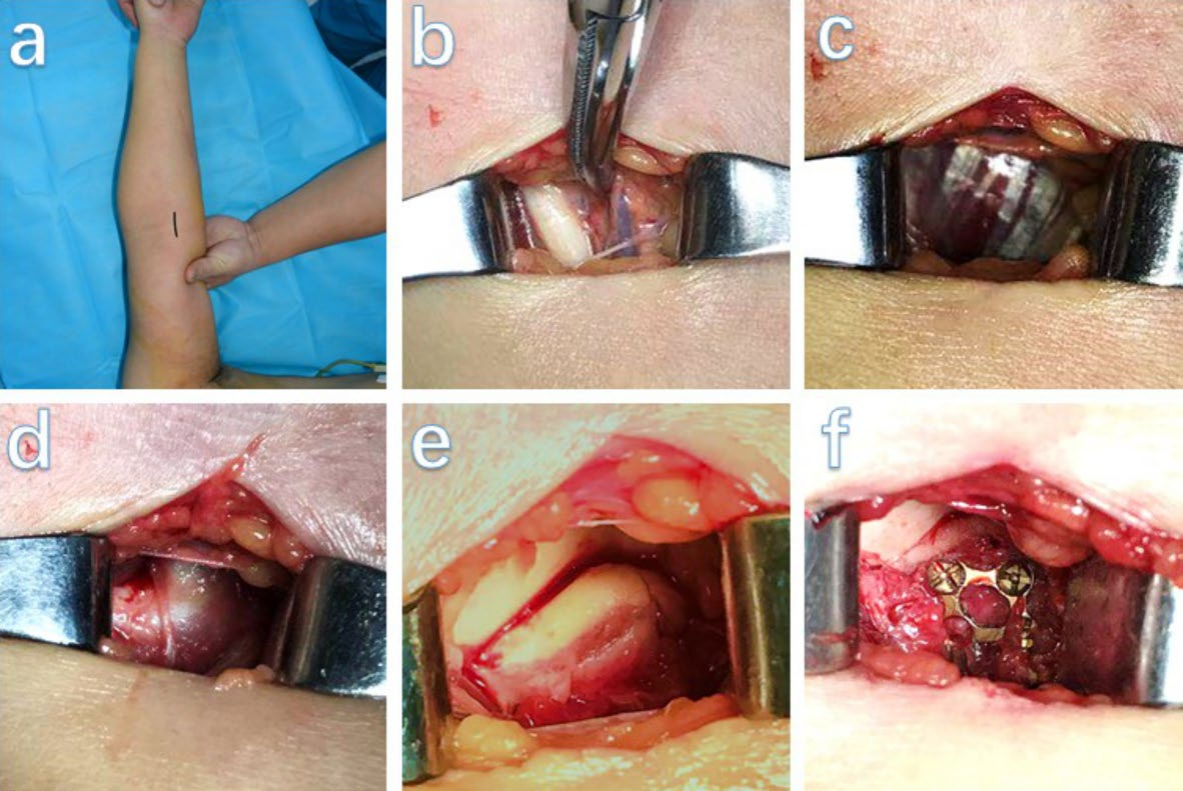
Representative of intraoperative photograph of coronoid fracture. (**a**) Straight anterior midline incision of the elbow. (**b**) Dissection between the biceps brachii tendon (white cords) and medial neurovascular bundle (brachial artery, vein, or nerve plexus). (**c**) Brachialis is located beneath the biceps brachii. (**d**) The anterior capsule of the elbow was revealed after dissecting the brachialis muscle. (**e**) The anterior capsule was carved longitudinally, and the fracture fragments are shown. (**f**) The fractured fragment was fixed using a mini-plate. The entire plate can be visualized by pulling the incision on the longitudinal side

A safe space was available between the brachial artery and the median nerve for insertion fixation devices. Intermuscular dissection between the brachial artery and the median nerve was performed. The brachial artery was retracted laterally, and the median nerve was retracted medially. We retracted the brachial artery, biceps tendon, and brachioradialis laterally, whereas the pronator teres and median nerve were retracted medially, which provided the best access to the brachial muscle. The brachialis muscle was longitudinally split to obtain a good exposure of the anterior capsule of elbow joint (Fig. 3).

The anterior joint capsule was incised for good visualization of the fracture site and the surface of the ulnohumeral joint. After hematoma evacuation and debriding of the fracture site surface, the fracture was reduced temporarily using a 1-mm Kirschner pin for fixation. After sufficient exposure to the fracture site, it was essential to confirm the patient’s bone quality and early functional recovery expectations to determine the number and position of the plate and fixation screw. Laterally, fixation was typically accomplished with a 2.7-mm locking mini-plate shaped according to the anterolateral morphology of the fracture site, which restored the height of the coronoid process. Several screws effectively fixed the coronoid fracture fragments. The surgeon needed to confirm that the screws did not penetrate the joint surface. When the internal fixation was complete, a layer-by-layer closure was performed. After definitive hemostasis, the tourniquet was released.

In patients with a terrible triad of elbow injury, the radial head fracture was exposed, and reduction and fixation was performed using the Kocher approach. An intraoperative valgus and varus stress test of elbow instability under fluoroscopy was performed; if the test was positive, the repair of the ligament of lateral collateral ligament (LCL) or the medial collateral ligament (MCL) with suture anchors was performed. Following fixation of the fracture and the repair of ligaments, the instability of the elbow was re-assessed under fluoroscopy. A hinged external fixator or orthosis was used for 3–5 days.

### Postoperative treatment and rehabilitation program

In accordance with the patient’s general condition, the patient initiated active exercising of the shoulder, wrist, and fingers from the day of surgery and active exercises of the elbow the day after pain and swelling had subsided, which generally required 3–5 days to subside. After suture removal, a fully active-assisted elbow ROM was initiated. Implant removal was not recommended for all patients because of the risk of neurovascular damage.

### Postoperative assessments

**Fig. 4:**
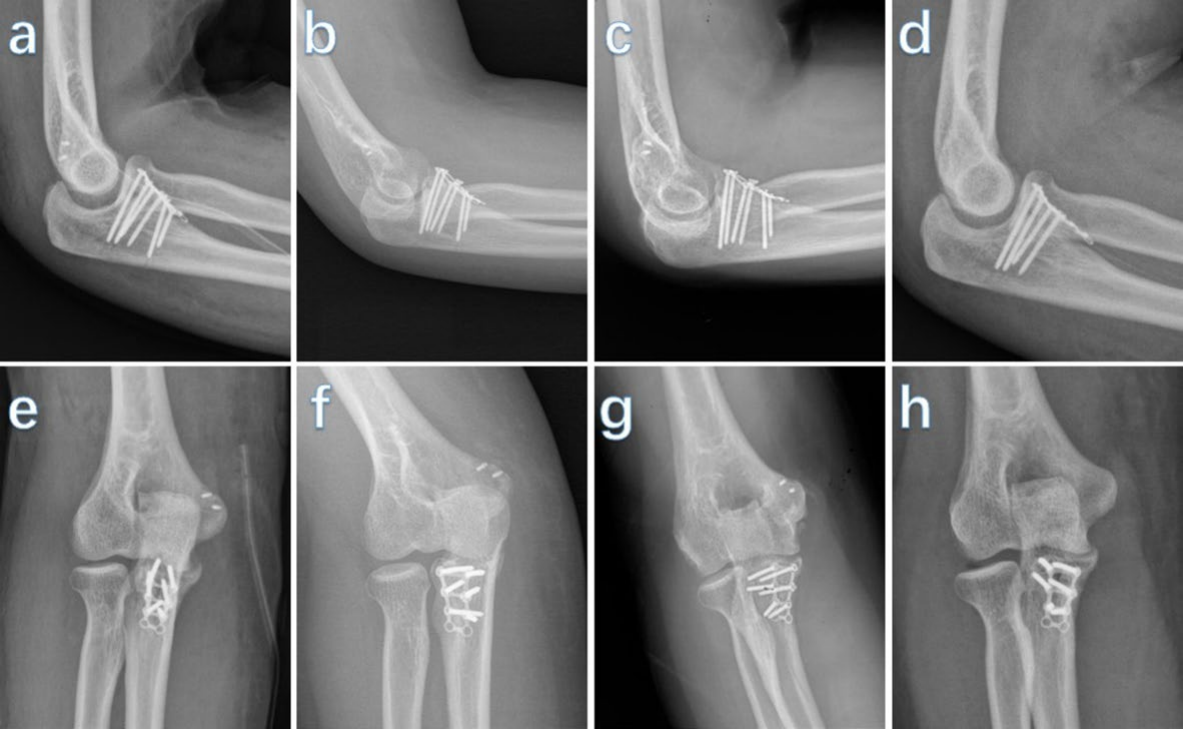
Radiographs obtained during follow-up. (**a**, **e**) After 4 weeks. (**b**, **f**) After three months. (**c**, **g**) After 6 months. (**d**, **h**) 12 months after the operation

**Fig. 5:**
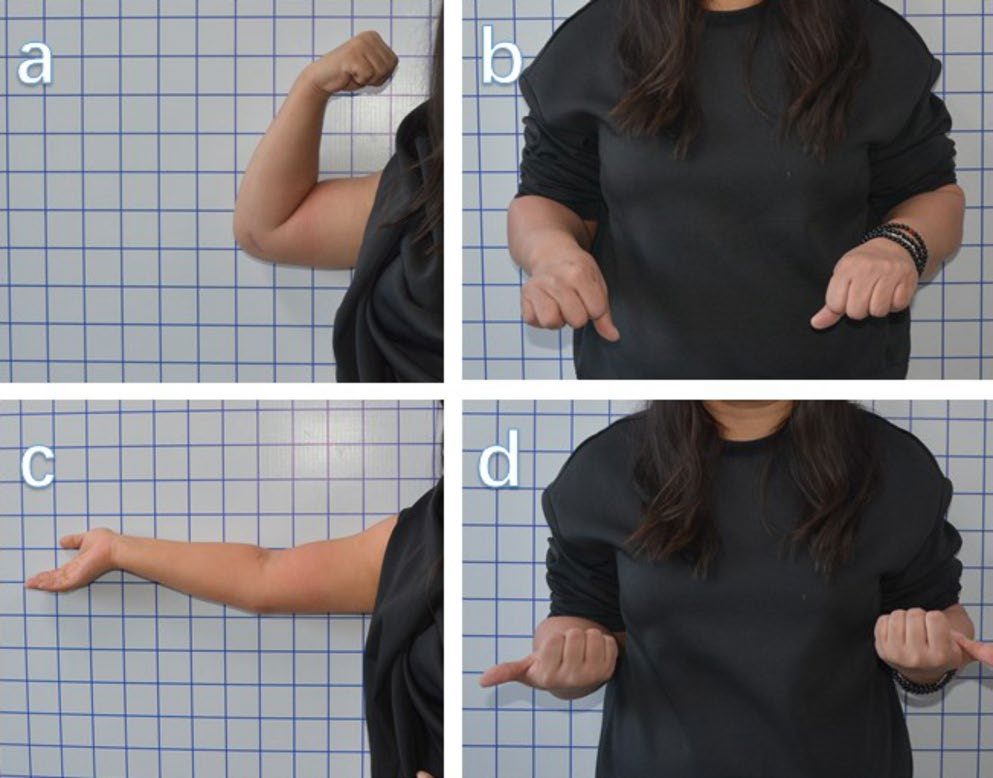
There was no significant limitation in flexion, extension, pronation, and supination of the left elbow at the 1-year follow-up. **a**) Flexion; **b**) pronation, **c**) extension, **d**) supination function of the injured limb

All patients underwent routine clinical and radiographic examinations (Fig. 4) and evaluations of the ROM at 1, 3, 6, and 12 months postoperatively and then annually. Complications, including nonunion, malunion, and HO, were recorded. During the follow-up, three doctors measured and analyzed each piece of data. The daily ROM of the elbow was evaluated using Morrey’s criteria ^9^ at the final follow-up (Fig. 5).

The Mayo Elbow Performance Index (MEPI) score was recorded postoperatively. The maximum score was 100 points; 90–100 was considered excellent, 75–89 was good, 60–74 was fair, and less than 60 was poor ^10^. Statistical analyses were not carried out because the study was retrospective and non-comparative.

## Results

### Clinical results

**Fig. 6:**
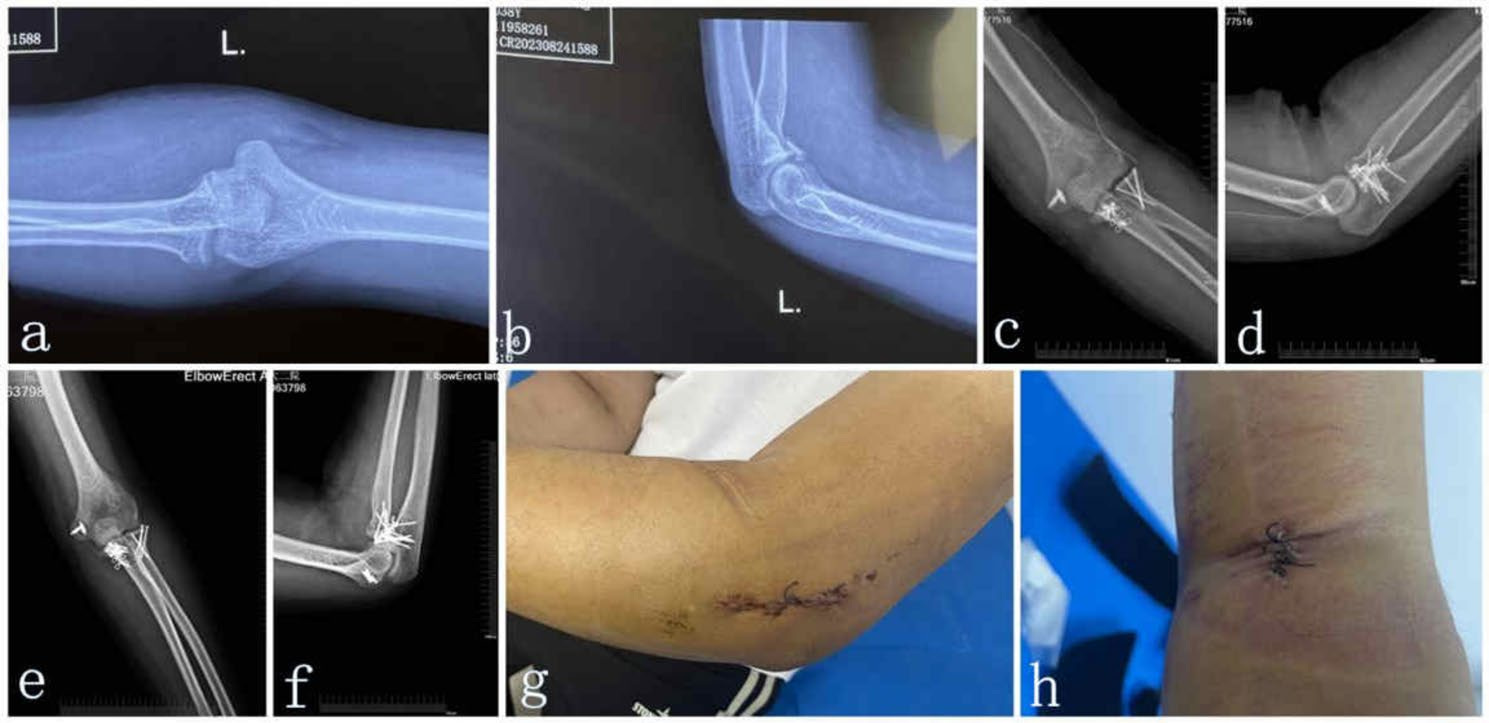
Representative “terrible triad injury of elbow”. (**a**, **b**) Preoperative radiographs. (**c**, **d**) Immediately postoperative radiographs. (**e**, **f**) After three months the operation. (**g**, **h**) The incision of the operation

All 32 elbows completed the short-term follow-up assessments for an average of 26.7 months (range, 19–40 months) (Table 2). *Of the total cases, 17 (53%) underwent isolated ulnar coronoid process fracture open reduction and internal fixation, while the others underwent ulnar coronoid process fracture open reduction and internal fixation combined with ligament repair. Of all the patients with ligament repair, 11 cases (34.3%) underwent LCL repair and 6 cases (18.7%) underwent MCL repair (*Fig. 6*).* The mean degrees of flexion, extension, pronation, and supination were 129.6 ± 4.6, 6.8 ± 2.9°, 81 ± 5.5°, and 83.9 ± 6.1 degrees, respectively. The final average VAS score was 0.8 ± 0.9 (range 1–3). According to Morrey’s criteria, 26 elbows (81%) achieved desired ROM after surgery. Elbow pain, ROM, stability, and function were comprehensively assessed using the MEPI, with a score of 93.1 points (range, 88–96), leading to excellent assessment for 26 cases (81.3%) and good assessment for 6 cases (18.7%) (Table 2).

**Table 2:** Perioperative parameters of the coronoid process fractures patients

Category	
Surgery time (minutes)	135 (range, 90–165)
Healing time (months)	3.6 (range, 3–5)
Radiographic evaluation	
Anatomic reduction	32
Poor reduction	0
Range of elbow daily motion (Morrey’s criteria)	
Achieved	26
One direction of motion below standard	6
Multiple directions of motion below standard	0
VAS score of last follow-up	2.1 (range, 1–3)
MEPI score	93.1 (range, 88–96)
Comprehensive assessment of MEPI	
Excellent	26
Good	6
Fair	0
Poor	0
Number of repair ligaments	
Isolated repair of LCL	11
Isolated repair of MCL	6

VAS, Visual analogue scale; MEPI, Mayo Elbow Performance Index; MCL, medial collateral ligament; LCL, lateral collateral ligament

### Radiological results

The union of coronoid processes was identified on radiological assessment after the operation. None of the cases experienced nonunion or delayed union, the failure of fixation devices, and the duration for the radiological union was 3.6 ± 1.3 months (Table 2).

### Complications

There were no intraoperative complications. None of the cases experienced elbow instability, joint stiffness, or dislocation. A total of 10 elbows (31%) experienced mild HO. Of these HO cases, 8 patients who underwent additional ligament repair during the open reduction internal fixation showed HO on the posterolateral, posteromedial, or anteromedial side, and the other elbows without ligament repair showed HO on the anteromedial side. None of the patients experienced nerve dysfunction, skin cutaneous necrosis, or wound infection (Table 3).

**Table 3:** Summary of complications

Category	
Nerve palsy	/
Ulnar nerve palsy	0
Median nerve palsy	0
Heterotopic ossification	
Asymptomatic	4
Symptomatic	6
Screw loosening and implant failure	0

## Discussion

The highly-extensile anterior minimally invasive approach to coronoid incision was used in this study to apply open reduction internal fixation with a mini-plate fixation for coronoid process fractures. Successful healing and good functional outcomes with surgical treatment were recorded for patients with surgical indications. Notably, our novel minimally invasive anterior approach produced favorable results.

Some soft tissue insertions on the coronoid include the anterior capsule and the anterior bundle of the medial ulnar collateral ligament [19, 20]. Sufficient fixation is required for most comminuted coronoid process fractures to allow early rehabilitation [21]. Anatomical reduction and strong fixation are necessary to treat anteromedial facet fractures of the coronoid process [22]. Our approach was based at the safe zone among the median nerve, brachial artery, and ulnar artery (Fig. 2). Due to the absence of significant branches across these regions [20], there was no need to cut any blood vessels to avoid causing damage. Through our approach, the fracture may be directly exposed, and a precise anatomical reduction and stable fixation without extensive dissection can be achieved, reducing complications such as nonunion, pain, stiffness, and HO, which is superior to conventional surgical methods.

Treatments for coronoid process fracture remain controversial in clinical practice. Various approaches to operative exposures for the complex coronoid process of fractures have been reported. The medial approach was previously regarded as the “gold standard” to treat isolated coronoid fractures [23, 24], but the method requires extensive exposure, similar to the lateral approach [25] or posterior approach [26] for coronoid fixation and capsular repair [27–29]. This approach entails high-risk of residual instability, neuro-palsy, and HO. Furthermore, the approach mentioned above does not perfectly expose the anterolateral or anteromedial coronoid fragment, making it difficult to repair the anterior joint capsule and the ligamentous insertion surrounding the coronoid.

Based on our findings in this study, a small anterior incision can efficiently complete reduction and fixation for patients with isolated ulnar coronoid fractures. However, our incision still presents some weaknesses for patients with dislocations or with the terrible triad. Additional medial or lateral incisions are necessary because of the need to complete the reduction and the fixation of the proximal radial fracture, as well as the repair of the medial or lateral collateral ligaments. Additional incisions mean increased operating time and increased bleeding.

The anterior minimally invasive approach had some benefits: First, it provides a direct visualization of the coronoid process fracture that permits satisfactory anatomical reduction of the coronoid and accurately places the plate based on the classification of the coronoid process fracture. Second, the anterior joint capsular and ligament attachment to the coronoid can be effortlessly repaired. When small coronoid fragments (Types I or II) accompany radial head fracture, this requires stabilization. Third, the mini-plate plays a role in helping the reduction of the fracture fragment. Tiny fracture fragments can be fixed with mini-plates, restoring the height of the coronoid process. The mini-plate can also act as a buttress plate for large fracture fragments. These features provide stable fracture fixation, which could lead to early and safe mobilization.

What is the best option for managing ulna coronoid process fractures? The fixation methods for coronoid processes fracture remains controversial. Several methods have been reported to fix coronoid process fractures, including a suture anchor, screw, plate, and steel wire suture. Morellato et al. reported that compared with plates, the isolated screw showed inferior stability for coronoid process fractures [30]. A study on treating coronoid fractures found that the mini-plate and screw have superior outcomes than the Kirschner wire and steel wire suture [31]. At our institution, we have elected to use this special mini-plate. Compared with T-shaped or Y-shaped fracture plates, two rows of 8-hole mini-plates are more suitable. The multi-screw structure helps to provide greater strength. This mini-plate can also be shaped according to the anteromedial facet of the ulnar coronoid process to effectively anatomical reduction.

In this study, 10 elbows (31%) experienced HO. The occurrence of HO is associated with certain predisposing conditions, such as orthopedic surgery, fractures, or dislocations, which may occur in up to 30% of elbow trauma and dislocations [32]. During the immobilization or rehabilitation exercise after an elbow joint injury, the elbow joint is usually in a 90° flexion position. When the shoulder joint is in a 90° abducted posture, the posterior medial side of the entire elbow joint is at the lowest point of the upper limb, and the hemorrhage from the fracture site gathers in this area, causing HO. Park et al. [33] evaluated 42 patients with limited ROM of the elbow due to an extrinsic contracture following trauma and found that more than 50% of cases of HO occur on the posteromedial side of the elbow, which is consistent with our findings. In addition, the failure to dissipate the local soft tissue hematoma caused by early rehabilitation exercise is also another reason.

This novel approach has potential drawbacks. For patients with the “terrible triad injury of elbow,” we recommend additional application of a lateral or medial incision along with a minimally invasive anterior approach to reconstruct the coracoid process and repair the radial head fracture, lateral or medial stabilizing complex, respectively. Although an additional incision is utilized, it reduces the incidence of complications by causing damage to the soft tissues due to extensive dissection to expose the fracture fragments using an isolated incision. The minimally invasive anterior approach has advantages of minimal intraoperative soft tissue damage, enhanced recovery after surgery, and high patient-reported satisfaction, but inevitably has a relatively long learning curve. For orthopedic surgeons, the learning curve can be shortened, and the incidence of complications reduced through strict screening of indications and cadaveric anatomy training.

Our study has some limitations. First is the small sample size. The small number of cases in this series might lead to higher variability, which may lead to bias. Second, this was a retrospective study without a control group from a single orthopedic center. Third, no comparison with other approaches was attempted. Thus, randomized controlled prospective studies with larger case numbers are necessary to evaluate different approaches further.

## Conclusions

To our knowledge, this retrospective study is the first to assess the application of reduction and mini-plate fixation of coronoid fractures using a highly-extensile approach. Based on our observations, this incision is feasible because it achieves anatomic reduction and high-efficiency fixation of the coronoid process. For the isolated comminuted coronoid process fracture with adequate elbow joint stability, the anterior minimally invasive approach avoids soft tissue invasion and allows superior exposure with a potentially lower risk of complications.

## Data Availability

The datasets are available from the corresponding author on reasonable request.

## Abbreviations

HO: Heterotopic ossification
MEPI: Mayo Elbow Performance Index score
ROM: Range of motion
VAS: Visual analog score
